# Phylogenetic analysis of *Arthrospira* strains from Ordos based on 16S rRNA

**DOI:** 10.1038/s41598-022-18695-7

**Published:** 2022-08-23

**Authors:** Jingnan Zhang, Huiting Xue, Sirui Yan, Haibo Shi, Ling Du, Junfeng Zhang, Da Huo, Ruiping Hu, Hong Yuan, Chunli Ma

**Affiliations:** 1 College of Basic Medicine, Inner Mongolia Medical University, Hohhot, 010110 China; 2 Inner Mongolia Third Hospital, Inner Mongolia Medical University, Hohhot, 010110 China; 3 College of Life Science and Technology, Inner Mongolia Normal University, Hohhot, 010022 China

**Keywords:** Genetics, Microbiology, Limnology

## Abstract

To study the growth rate of different *Arthrospira* strains, three species of *Arthrospira* from Ordos alkaline lake, labeled as sp.DD, sp.ER, sp.FB, one species of *Arthrospira* from Hasu Sea in Hohhot, labeled as sp.HS, another purified strain labeled as sp.QD donated by the Ocean University of China had been collected. The first four need to be further isolated and purified in culture. The growth curves of all strains were plotted. Subsequently, 16S rRNA sequences were amplified and sequenced in an attempt to study taxonomic relationships. The results showed that the growth rate was increased in the first 9 days, and sp.DD had the highest growth rate. Analysis of the sequencing results revealed that sp.HS had 99.79% homology with *Arthrospira platensis strain Sp-2*, sp.DD had 99.69% homology with *Arthrospira platensis FACHB834*, sp.QD had 99.54% homology with *Arthrospira platensis F3S*, sp.ER had 99.79% homology with *Arthrospira erdosensis *‘*Inner Mongolia*’, sp.FB had 99.74% homology with *Arthrospira erdosensis *‘*Inner Mongolia*’. Phylogenetic analysis indicated that sp.HS was closely related to *Arthrospira platensis strain Sp-2*; sp.DD and sp.QD had a close genetic relationship; sp.ER and sp.FB had a close genetic relationship. In conclusion, these findings provide a theoretical basis for the further development and reproduction of dominant algae species in Inner Mongolia through biological analysis of *Arthrospira*.

## Introduction

*Arthrospira* is an importantly commercial filamentous cyanobacteria and the largest kind of microalgae^1^. *Arthrospira platensis* and *Arthrospira maxima* are the most commonly commercial application, generally named as incorrect brand name ‘*Spirulina*’. Living in tropical and subtropical warm lakes with a high carbonate and bicarbonate content, and high pH and salinity^2^, *Arthrospira* is rich in proteins, minerals, vitamin B_12_, β-carotene, and essential fatty acids, such as γ-linolenic acid which can inhibit bacteria and virus, regulate the immune function, and can prevent diseases like hypertension, diabetes and tumor, etc.^3^. Thanks to its high protein content of up to approximately 60–70% on a dry weight, the amino acids present in *Arthrospira* match the proportions recommended by the Food and Agriculture Organization (FAO)^4^. Besides, it does not require any chemical or physical processing in order to become digestible due to the absence of a cellulose cell wall^5^.

Microscopic examination for cyanobacteria is rapid and sensitive. However, even for skilled and experienced operators, sometimes it is impossible to determine the identity. Thus, molecular classifications help to detect and identify specific strains, especially strains with the same morphology at the species level, such as species in cyanobacterial genera, including *Microcystis*, *Anabaena*, *Nodularia*, and *Cylindrospermopsis*^6,7^. During recent decades, phylogenetic studies have been frequently used in the taxonomy of *Arthrospira* strains, including 16S rRNA^8^, 16S–23S rRNA ITS^9,10^, cpcBA-IGS^8^, and some other regions of the genome^11,12^. 16S rRNA has large information to reflect biological evolutionary processes, which is the most commonly used for the identification of biological classification at present^13,14^.

Through investigating the spots of eight alkalines of Maowusu Sandy Land and Kubuqi Desert of Ordos Plateau in Inner Mongolia, four species of *Arthrospira* stains were found, among which two of them were new species and the other two were newly recorded in China^15^. One of them was *Arthrospira platensis*, which has been applied to industrialized cultivation. It was a natural algae species adapted to low-temperature and broad temperature^16^. In view of the ecological advantages of alkali lake in Ordos, the Etok Industrial Park in Ordos has been established by using local materials, which is the largest Spirulina culture base in China. Then we amplified the 16S rRNA sequence for identification to contribute to germplasm resource protection and development of *Arthrospira* strains from Ordos in Inner Mongolia.

## Materials and methods

### Organisms and culture conditions

**Figure 1:**
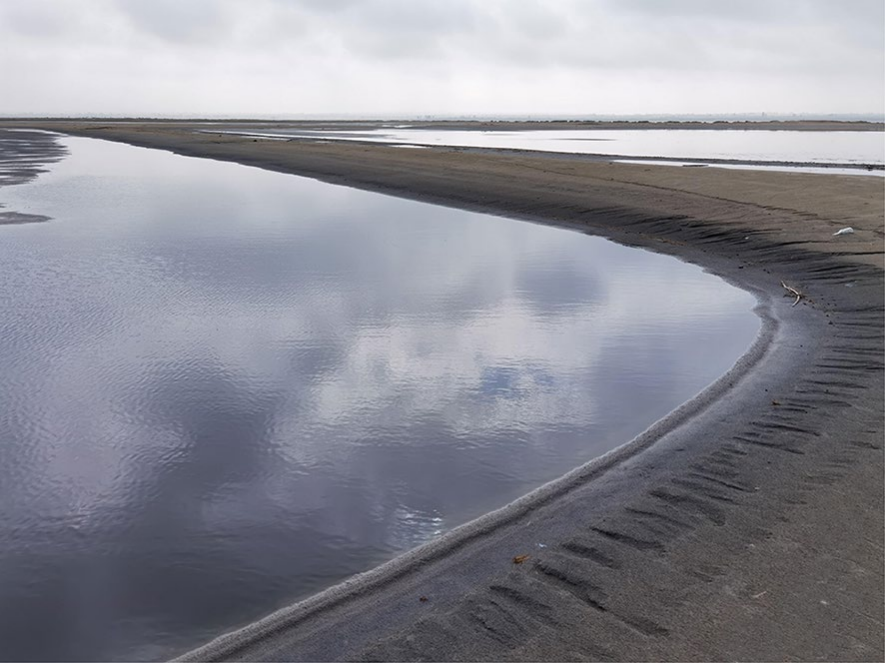
*Arthrospira* strains found in Bayannur Lake. Retrieved from: https://www.meipian6.cn/38baf2jz.

**Figure 2:**
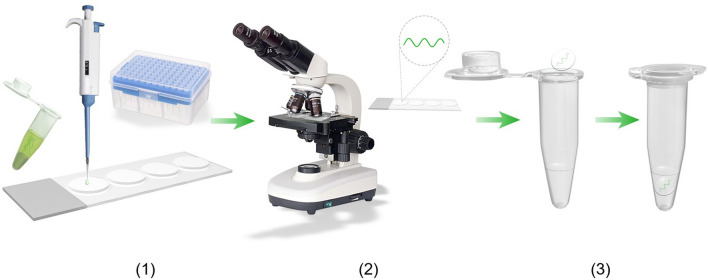
The progress of separating and purifying the five *Arthrospira* strains. Made by 3dmax (2020) and Photoshop cc (2020).

There are five *Arthrospira* strains studied in this article, three of which were collected from Ordos alkaline lake (39°14′N, 108°04′E) (Fig. 1), respectively labelled as sp.DD, sp.ER, and sp.FB; one of which was collected from Hasu Sea in Hohhot (41°02′N, 111°39′E), labelled as sp.HS; and one of which was donated by Ocean University of China, labelled as sp.QD. The five *Arthrospira* species were preserved in natural light and at room temperature, then separated and purified as follows, and the progress was shown in Fig. 2.

(1) Unpurified algae were diluted with sterilized Zarrouk medium (at 1:5 dilution). Four sterilized cell climbing slides (5 mm) were placed equidistantly on sterilized slides, and 1 μL of diluted solution was quickly dropped onto the cell slides. (2) Four cell climbing slides were observed under the low magnification to make sure in which there was a single cyanobacterium. If the target cyanobacterium was found, observe it under the high magnification to confirm there were no other contaminants for example *Oscillatoria* and Brine Shrimp. If not, the algae medium was diluted more times until a single alga was obtained. (3) The cell slides with the target alga were transferred to an Eppendorf tube containing 100 μL of Zarrouk medium. When the algal liquid turned green, the microscopic examination was again carried out. When there were no plankton and other algae species in the culture medium, the purified algae species were obtained.

The morphological characteristics were observed using a Leica ICC50 HD microscope (Leica, Germany) when the cultures were in their exponential growth phase.

### Plot the growth curve

An appropriate amount of algae liquid of expanded culture was collected, centrifuged at 3000 r/min for 5 min, followed with discarding the supernatant. Algae sediments were resuspended and washed with sterilized Zarrouk medium for three times. Then algae sediments were inoculated into 100 mL triangular flask by adjusting the concentration of algae liquid with sterilized Zarrouk medium, in order to make the initial A_560nm_ = 0.05 in a total volume of 50 mL, and placed it in natural light and at room temperature, with shaking it twice a day. 100 μL of algae liquid was sampled at 10 o'clock everyday to measure A_560nm_ using a Synergy LX Multimode Reader (Agilent BioTek, America), and the experimental cycle was 20 days. Plot the growth curve with days as X axis and A_560nm_ as Y axis.

### Phylogenetic analysis

An appropriate amount of algae liquid were collected by centrifugation (12,000 r/min, 4 °C, 5 min) and washed twice with sterile distilled deionized H_2_O. Genomic DNA was extracted using a Plant Genomic DNA kit (Tiangen, Beijing, China). Finally, the DNA was dissolved in 100 μL of buffer TE and stored at − 20 °C. PCR was carried out on the DNA extracts using the following primer pairs FP (5′-AGAGTTTGATCCTGGCTCAG-3′) and RP (5′-TTTGCGGCCGCTCTGTGTGCCTAGGTATCC-3′)^17^ for the amplification region of 16S rRNA and 16S–23S rRNA-ITS. The PCR mixture consisted of: 9.5 μL of nuclease-free water, 12.5 μL of 2 × Taq Master Mix, 0.5 μL of each primer, and 1 μL of genomic DNA in a total volume of 24 μL. The PCR conditions were: denaturation for 5 min at 94 °C, followed by 35 cycles of amplification for 1 min at 94 °C, 1.5 min at 55 °C and 3.5 min at 72 °C followed by a final elongation step of 7 min at 72 °C. Thermal cycling was carried out using a LongGene A300 PCR Amplifier (LongGene, Hangzhou, China). PCR products were visualized by 1.5% (w/v) agarose gel electrophoresis in 1 × TBE buffer and GeneRed Advanced DNA staining (Tiangen, Beijing, China).under UV transillumination and were sequenced by RuiBiotech Biotechnology Co. Ltd. (Qingdao, China). Multiple sequence alignment and phylogenetic tree construction of each locus were carried out using the MEGA11 software^18^. A phylogenetic tree was constructed by applying the Maximum-Likelihood method^19^ with the distance matrix to be computed using the Tamura-Nei Model^20^ for dissimilarity values. Bootstrap analysis was also performed in a total of 1000 replicates for the ML analysis.

## Results

### Morphological character

**Figure 3:**
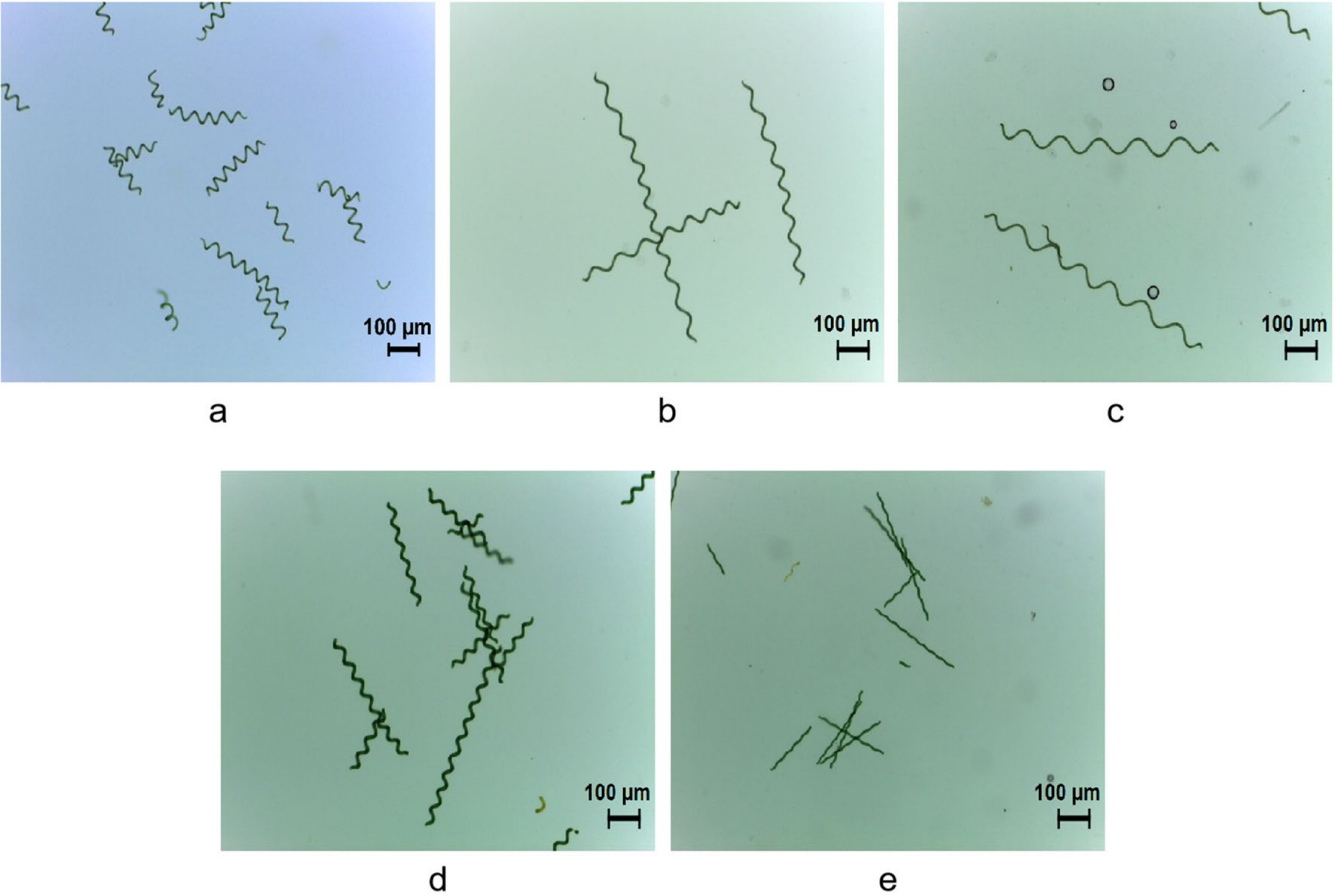
Photomicrographs of the five *Arthrospira* strains. (**a**) sp.HS; (**b**) sp.DD; (**c**) sp.QD; (**d**) sp.ER; (**e**) sp.FB.

The photomicrographs of the five *Arthrospira* strains were shown in Fig. 3.

### Growth curves of the five species

**Figure 4:**
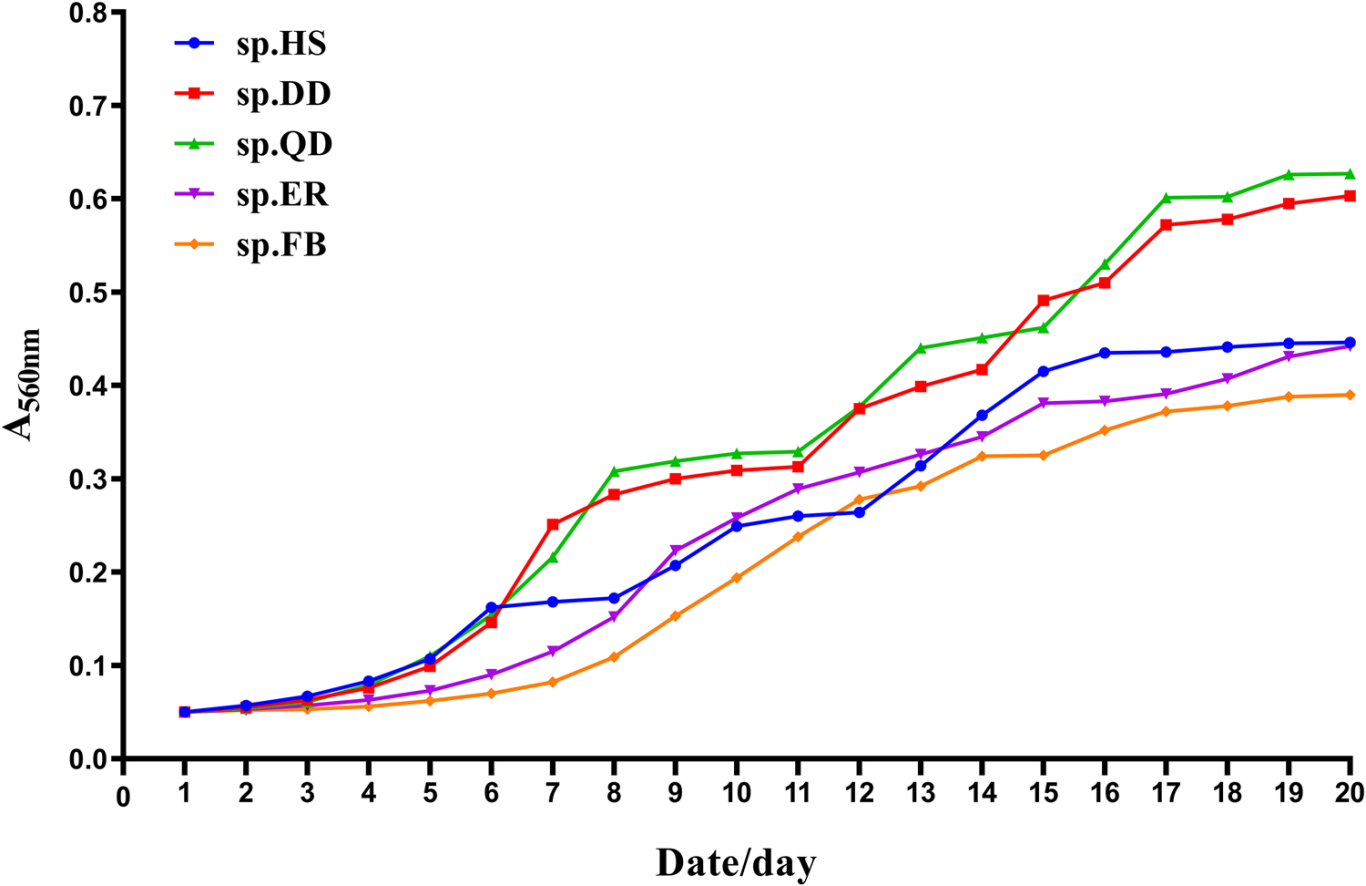
Growth curves of the *Arthrospira* five species.

The growth curves of the five species were shown in Fig. 4. As a whole, the trends of all the growth curves were similar, which were growth sharply at the early growth stage, then the rise slowed down. For sp.HS, the growth rate reached the top in the first 6 days, showing an exponential trend. For sp.DD, sp.QD, it was fastest in the first 7 days and 8 days. sp.ER and sp.FB had the fastest growth rate in the first 9 days and increased exponentially. The growth rate of sp.DD and sp.QD was higher than that of the other three, while the maximum growth rate of sp.DD was higher than that of sp.QD.

### Phylogenetic analysis

**Figure 5:**
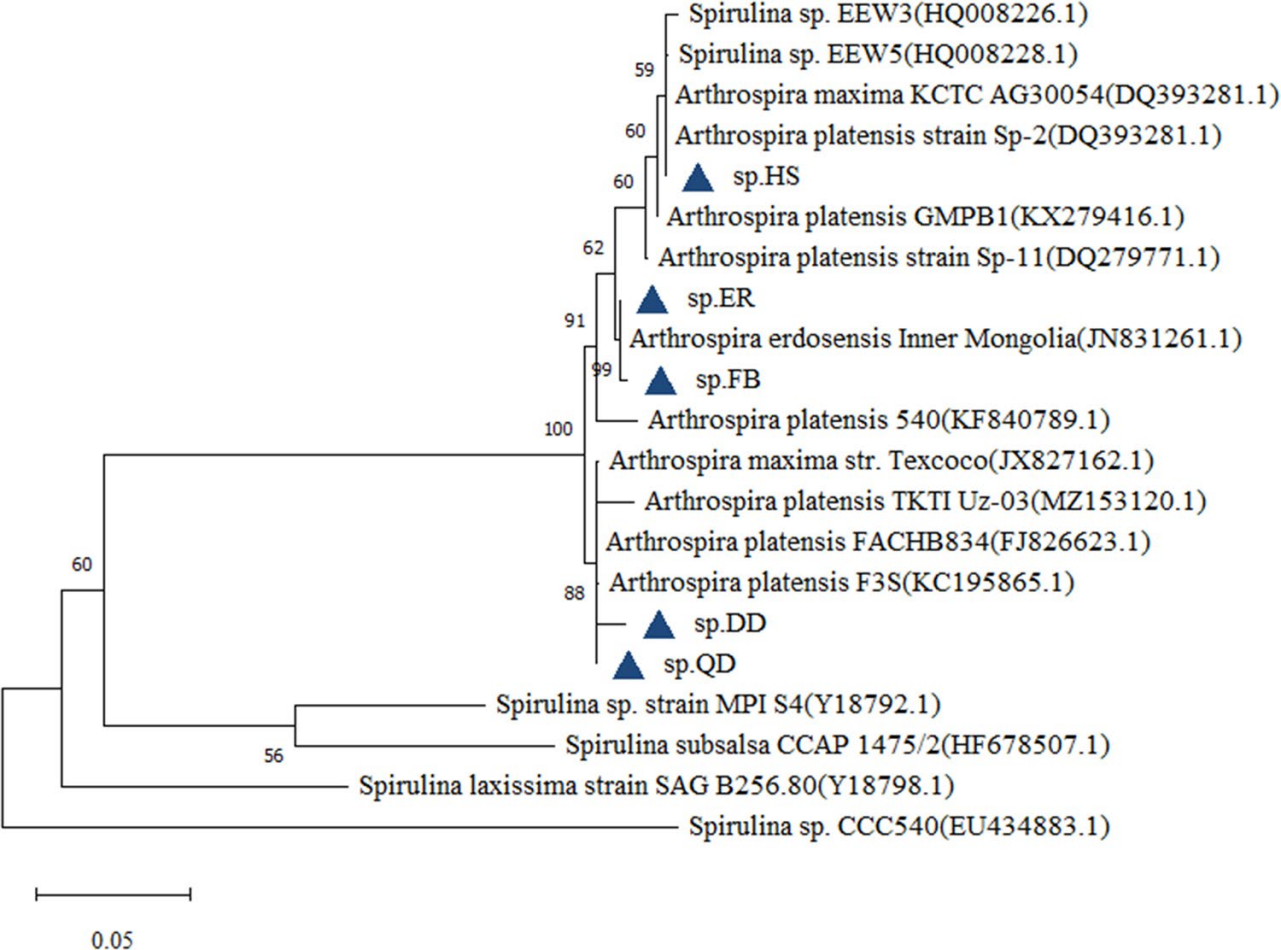
Molecular Phylogenetic analysis based on 16S rRNA-ITS by Maximum Likelihood method.

A nearly complete 16S rRNA and 16S–23S rRNA ITS gene sequence (from 1903 to 1934 nt) of five strains were obtained. Analyses of the five sequences produced strong similarities, totally above 99% (Table 1). Results of the 16S rRNA gene sequence global alignment in the NCBI database demonstrated that sp.HS had the highest similarity to *Arthrospira platensis strain Sp-2*; sp.DD had the highest similarity to *Arthrospira platensis FACHB834*; sp.QD had the highest similarity to *Arthrospira platensis F3S*; sp.ER had the highest similarity to *Arthrospira erdosensis* ‘*Inner Mongolia*’; sp.FB had the highest similarity to *Arthrospira erdosensis* ‘*Inner Mongolia*’. The phylogenetic tree (Fig. 5) constructed by the maximum-likelihood algorithm indicated that sp.HS was closely related to *Arthrospira platensis strain Sp-2*; sp.DD, sp.QD, *Arthrospira platensis FACHB834* together with *Arthrospira platensis F3S* clustered in the same branch and had a close genetic relationship. There was a close genetic relationship among sp.ER, sp.FB and *Arthrospira erdosensis* ‘*Inner Mongolia*’.

**Table 1:** Results of blastn in the GenBank database.

Strains name	Similarity (%)	Homologue	GenBank accession number
sp.HS	99.79	*Arthrospira* *platensis* strain Sp-2	DQ279768.1
sp.DD	99.69	*Arthrospira* *platensis* FACHB834	FJ826623.1
sp.QD	99.54	*Arthrospira* *platensis* F3S	KC195865.1
sp.ER	99.79	*Arthrospira* *erdosensis* ‘Inner Mongolia’	JN831261.1
sp.FB	99.74	*Arthrospira* *erdosensis* ‘Inner Mongolia’	JN831261.1

## Discussion

Our previous study found that the optimal growth temperature of sp.DD was 24 °C, which could maintain growth within the temperature range from 4 to 40 °C, and sp.DD could reproduce after treatment at − 18 °C for 24 h, which showed better adaptability to low temperature than *Arthrospira platensis* from Lake Chad^21^. The photosynthetic pigment content of sp.DD was ranked as phycobilin > chlorophyllin A > carotenoid^22^, and the total photosynthetic pigment content and phycobilin content of sp.DD were higher than those of *Arthrospira platensis* from Lake Chad^23^. Then sp.DD is more suitable for cultivation in northern China than the imported species, which has been used as one of the industrial algae species in Inner Mongolia.

The growth rates of all the five algae reached the peak in the early stage of culture and then decreased, which was related to the gradual consumption of nutrients in the medium. sp.DD had a faster growth rate and could achieve a higher bioaccumulation at the same time. Besause of the fastest growth rate of the first 7 days of sp.DD, it is compliant in industrialization. It would be better to supplement the culture medium on the seventh day for a higher growth rate and for more bioaccumulation.

The classical classification and phylogenetic studies of cyanobacteria such as *Arthrospira* are based on morphological characteristics, which are the basis of modern biological classification and phylogenetic studies. Biological morphology as a phenotype is the result of the interaction between genes and the environment. As the main morphological parameters of classical classification, the length, spiral number, pitch, and cell size of *Arthrospira* filaments may vary not only from species to species but also from the same species in different environmental conditions^24^. Vonshak^25^ reported that when the culture temperature increased and the light intensity increased, the spiral pitch of *Arthrospira* decreased, while cutting down the light intensity, the pitch could be very long. Bai^26^ described three typical *Arthrospira* forms and pointed out that these three forms could change with each other due to changes in the environment or the composition of the culture medium. Therefore, the classification of *Arthrospira* species should be based on morphological characteristics combined with physiological and ecological features, especially modern molecular biology and other research achievements and techniques. According to the 16S rRNA sequence, sp.HS, sp.DD, and sp.QD were separated as different strains of *Arthrospira platensis.* While sp.ER and sp.FB were *Arthrospira erdosensis* not being distinguished in the light of 16S rRNA sequence. A single sequence cannot accurately identify the strain, so it is necessary to use multiple genes to identify and classify the species. In the study of molecular systematics, it is necessary to search for more accurate molecular markers or improve the original molecular markers to adapt to the study of low order genetic relationships.

Germplasm resources are the basis of microalgae industrialization. Through screening and cultivating series of excellent microalgae species resources, the large-scale and high cultivation of microalgae can be improved and the diversity of commercial varieties can be increased. Wang et al.^27^ reported that when the initial Cu^2+^ concentration was in the range of 0.5–1.5 mg/L, a slight increase in the growth rate of FACHB-834 was observed. In contrast, when Cu^2+^ concentration was at or higher than 2.0 or 6.0 mg/L, respectively, the growth of FACHB-834 was inhibited and displayed to be yellow and fragmentation of filaments. *Arthrospira platensis FACHB-834*^28^ was cultivated in a modified Zarrouk medium supplemented with selenium. When selenium at low concentrations (1 mg/L, 10 mg/L) promoted the growth; its biomass increased significantly by 21% and 15% (*p* < 0.05), the maximum biomass reached 1.04 g/L at 1 mg/L of selenium. Liu^29^ reported that the photosynthesis of *Arthrospira erdosensis* took place in PSI and PSII systems and the diurnal variation of photosynthetic rate of *Arthrospira erdosensis* was a single-peak curve, and it did not show the “nap” phenomenon of some land plants. The application of characteristic algae species can not only meet the demand for high-quality microalgae species resources in algae science and other research fields, but also guarantee and promote the healthy and efficient development and industrial upgrading of microalgae industrialization, making further efforts to provide scientific and technological support for the basic research and industrialization of algae.

There were differences in long-term evolutionary status and phylogenetic relationship between *Arthrospira* strains in different ecological environments, which showed genetic diversity and germplasm resource diversity. In this study, *Arthrospira* species from Ordos in Inner Mongolia were identified for providing basic data for germplasm resource protection and development.

## Data Availability

The 16S ribosomal RNA gene, partial sequence; 16S–23S ribosomal RNA intergenic spacer, complete sequence; and 23S ribosomal RNA gene, partial sequence of *Arthrospira* platensis HS was submitted to GenBank (ON775378). The 16S ribosomal RNA gene, partial sequence; 16S–23S ribosomal RNA intergenic spacer, complete sequence; and 23S ribosomal RNA gene, partial sequence of *Arthrospira* platensis DD was submitted to GenBank (ON775379). The 16S ribosomal RNA gene, partial sequence; 16S–23S ribosomal RNA intergenic spacer, complete sequence; and 23S ribosomal RNA gene, partial sequence of *Arthrospira* platensis QD was submitted to GenBank (ON775380). The 16S ribosomal RNA gene, partial sequence; 16S–23S ribosomal RNA intergenic spacer, complete sequence; and 23S ribosomal RNA gene, partial sequence of *Arthrospira* erdosensis ER was submitted to GenBank (ON775381).
